# Parasitic Infections Associated with Unfavourable Outcomes in Transplant Recipients

**DOI:** 10.3390/medicina54020027

**Published:** 2018-05-01

**Authors:** Wojciech Wołyniec, Małgorzata Sulima, Marcin Renke, Alicja Dębska-Ślizień

**Affiliations:** 1 Department of Occupational, Metabolic and Internal Diseases, Institute of Maritime and Tropical Medicine, Medical University of Gdansk, 80–210 Gdansk, Poland; mrenke@gumed.edu.pl; 2 Department of Tropical and Parasitic Diseases, Institute of Maritime and Tropical Medicine, 81–519 Gdynia, Poland; m.sulima@poczta.fm; 3 Department of Nephrology, Transplantology and Internal Diseases, Medical University of Gdansk, 80–210 Gdansk, Poland; adeb@gumed.edu.pl

**Keywords:** infection, immunosuppression, tropics

## Abstract

*Introduction*. The immunosuppression used after transplantation (Tx) is associated with an increased risk of opportunistic infections. In Europe, parasitic infections after Tx are much less common than viral, bacterial and fungal ones. However, diseases caused by parasites are very common in tropical countries. In the last years the number of travellers with immunosuppression visiting tropical countries has increased. *Methods*. We performed a literature review to evaluate a risk of parasitic infections after Tx in Europe. *Results*. There is a real risk of parasitic infection in patients after Tx travelling to tropical countries. Malaria, leishmaniasis, strongyloidiasis and schistosomiasis are the most dangerous and relatively common. Although the incidence of these tropical infections after Tx has not increased, the course of disease could be fatal. There are also some cosmopolitan parasitic infections dangerous for patients after Tx. The greatest threat in Europe is toxoplasmosis, especially in heart and bone marrow recipients. The most severe manifestations of toxoplasmosis are myocarditis, encephalitis and disseminated disease. Diarrhoea is one of the most common symptoms of parasitic infection. In Europe the most prevalent pathogens causing diarrhoea are *Giardia duodenalis* and *Cryptosporidium*. *Conclusions*. Solid organ and bone marrow transplantations, blood transfusions and immunosuppressive treatment are associated with a small but real risk of parasitic infections in European citizens. In patients with severe parasitic infection, i.e., those with lung or brain involvement or a disseminated disease, the progression is very rapid and the prognosis is bad. Establishing a diagnosis before the patient’s death is challenging.

## 1. Introduction

The number of immunocompromised patients after solid organ transplantation (SOTx) exceeded one million in 2004 [1]. About 120,000 solid organs are transplanted annually [2]. There are also growing populations of patients after bone marrow transplantation (BMTx), patients with human immunodeficiency virus (HIV) infection and those treated with chemotherapy or new monoclonal anti-bodies.

The transplantations (Tx) and other expensive medical procedures are performed mainly in developed countries, and parasite infections are typical for the developing world, where about three billion people are infected by at least one of the 342 human parasites [1]. However, sometimes these “two worlds” meet. A good example is Brazil, a predominantly tropical country, which performs the second-highest number of kidney (KTx) and liver (LTx) transplantations in the world [3]. Epidemiological information concerning parasitic infections after Tx seems to be inadequate. The true incidence of post-transplantation parasitic infections (PTPI) remains unknown [1]. This review deals mainly with the problem of PTPI in Europe.

## 2. Methods

We performed a literature review to evaluate the risk of parasitic infections after Tx in Europe. A literature search was conducted and all relevant articles published until March 2017 identified from the Medline and PubMed databases were retrieved. The key terms used in the search strategy included ‘parasite OR parasitic’ AND ‘transplant OR transplantation OR graft’. Moreover, secondary searches of the reference lists in the included peer-reviewed articles were conducted to identify additional eligible studies. The search was limited to English language publications.

## 3. Results

### 3.1. Classification of Parasitic Infections

Parasites are generally divided into three main classes: protozoa, helminths and ectoparasites. Protozoan infections attract the greatest attention due to the highest mortality observed in this group of pathogens. One of the most life-threatening is *Naegleria fowleri*, with a fatality rate ranging from 95 to 99% [4].

Generally, there are two clinical pictures of parasitic infections observed after Tx. The first one is acute systemic illness, with anaemia, fever, multiorgan involvement and their failure. These symptoms are similar to septic shock. Many parasitic infections, especially protozoan infections and strongyloidiasis, can provoke such disseminated disease [5]. The second manifestation is correlated with local symptoms, most frequently gastrointestinal (GI) disorders such as diarrhoea and abdominal pain. Some patients present severe neurological signs, eye involvement, skin changes or urinary symptoms [5].

### 3.2. The Evidence of Infections in Europe

In contrast to tropical areas, severe parasitic infections are uncommon in Europe. The incidence of life-threatening tropical parasitic disease—malaria was 1.0 case per 100,000 population/year in 2015. The incidence rates for individual countries varied between 0.1 cases (Hungary, Latvia, Poland) and 2.6 cases (Sweden) per 100,000 per year [6]. The incidence rate of alveolar echinococcosis, associated with Europe and the Northern Hemisphere is only 0.2 cases per 100,000 population per year [7]. Toxoplasmosis is common, but most of the cases are asymptomatic in the adult population. Nevertheless, factors such as climate change, migration and globalization can alter this “benign” epidemiology. Moreover, the population of Europe is ageing and the number of immunosuppressed patients is increasing. 

### 3.3. Risk Factors

Travel to tropical countries and immunosuppression (IS) seem to be the main risk factors of PTPI. Most of the parasites are present only in endemic areas, therefore the most common type of parasitic infection after transplantation in Europe is **a new-onset disease in travellers** (Table 1) [1].

The **reactivation of the disease** is possible mainly in immigrants and patients who have visited the tropics and subtropics before transplantation. The reactivation of a cosmopolitan disease, like toxoplasmosis or echinococcosis is also possible. Some infections can remain asymptomatic for many years before IS implementation. In Europe the reactivation of latent toxoplasmosis and tropical diseases is observed after SOTx [8]. In tropical countries persistent and relapsing infections are typical of malaria, leishmaniasis, strongyloidiasis and others [1,9].

**Donor-derived disease** is less common. The transmission of malaria, Chagas disease, toxoplasmosis and leishmaniasis with transplanted organ have been described [1]. In Europe this type of transmission is uncommon (Table 2).

### 3.4. Major Parasitic Infections in Transplant Patients

**Malaria** is one of the most common diseases worldwide. According to Borosum, an Egyptian specialist on parasitic nephropathies, “the life cycle of this protozoan is ideal for becoming a notorious post-transplant infection” [1]. Typical symptoms are fever and anaemia [1]. The most severe clinical courses of malaria are caused by *P. falciparum* [10]. The mortality in the immunocompetent population is as low as 0.25% [1]. Nevertheless, because of the high number of infected patients, this single disease causes more than 400,000 deaths a year (2013) [11]. Malaria incidence is not increased compared to a healthy population [12], but mortality can reach 40% in liver and 6% in kidney recipients [13]. Post-transplant malaria is an insidious disease without the typical periodicity of fever with severe anaemia, thrombocytopaenia, acute respiratory and graft failure [1,10]. New-onset disease caused by the bite of the *Anophele* mosquito is the most frequent way in which malaria is acquired in immunocompromised individuals who travel to the tropics [10]. Cases of malaria transmitted by blood or organ transplantation have been reported mainly in Africa, but also in the USA and Europe [3]. The reason for this is that in its life-cycle *Plasmodium* sp. is present within red blood cells and hepatocytes. In endemic countries, *Plasmodium* sp. could be transferred with solid organ or bone marrow transplants [1,14]. *P. falciparum* can survive at the standard graft preservation temperature (40 °C) for up to 24 h [13]. There is also a risk of the reactivation of malaria in endemic countries. The reactivation of malaria is possible in patients with a history of *P. vivax* or *P. ovale* infection, because dormant forms of these species, hypnosoides, can survive for up to four years in the liver [1].

Prophylaxis is strongly recommended for travellers. The best way is to use chemoprophylaxis and long lasting insecticide-impregnated bed nets [10]. However, routine chemoprophylaxis is not recommended for recipients staying longer or living in endemic countries [12]. There are problems with drug interactions between IS and the agents used for malaria prophylaxis and treatment. Mefloquine, doxycycline, chloroquine and primaquine may increase calcineurin inhibitor levels. Sulphadoxine-pyrimathamine and quinine sulphate may decrease their levels. Doxycycline reduces the level of mycophenolate [15].

**Leishmaniasis** is a vector-borne disease transmitted by a sand-fly, an insect that can be encountered in about 88 countries in the tropics and subtropics as well as in southern Europe [10,16]. Human infections are caused by about 21 species of *Leishmania*. The clinical manifestations are: visceral (VL, kala azar), cutaneous (CL) and mucocutaneous (MCL) [3]. Around 500,000 new cases of VL and 5000 deaths caused by VL are observed annually. VL is endemic in more than 60 countries, with most cases of VL reported in India, Bangladesh, Sudan, South Sudan, Brazil, Ethiopia and Nepal [3,17,18,19]. VL is also diagnosed in the southeast of the USA, China and the Mediterranean basin [16] In Europe 340–510 cases are diagnosed annually [20]. The most typical and common symptoms of VL are irregular fever, hepatomegaly, splenomegaly, weight loss and pancytopaenia [19]. Most immunocompetent patients will not develop disease after *Leishmania* infection [16]. In some highly endemic areas up to 30% of inhabitants are asymptomatically infected [20]. The symptoms of disease can occur as late as 30 years after an initial infection [16].

The incidence of symptomatic disease is higher in immunocompromised patients. In Spain, the incidence of VL in HIV-positive individuals is 418-fold higher than in HIV-negative individuals [17,20]. In immunocompromised patients severe disseminated disease with pulmonary and kidney involvement was observed [10,16,17]. About 100 cases of VL after Tx were reported in the literature, with a mortality rate of about 20% [16]. A possible means of transmission from person to person is through injected blood or contaminated syringes, or through infected grafts [17,18,19]. Although newly-acquired disease prevails after transplantation, more than 50 cases of the reactivation of latent infection have been described in the literature [1]. Transmission through infected organs during Tx has been suggested [1,17]. 

Tissue specimens and peripheral blood, bone marrow or liver stained aspirate smears may be analysed for the presence of macrophages filled with amastigotes. Serologic detection of antibodies to recombinant K39 antigens of *Leishmania* and polymerase chain reaction (PCR) assay are highly sensitive and specific in VL diagnosis [16]. Protective means to prevent exposure to sand-fly bites are recommended. Serological screening of transplant recipients with a history of potential exposure to *Leishmania* may be considered before Tx [20].

In CL, typical ulcerative skin lesions are present at the site of the sand-fly bite. In many cases, CL heals spontaneously leaving scars [20]. Severe clinical forms of CL and MCL with parasite dissemination were reported in patients after Tx [17,19]. Skin and mucosal changes in patients returning from endemic areas should always be suspected of CL or MCL [20].

**Toxoplasmosis** is a cosmopolitan, world-wide distributed, opportunistic zoonosis transmitted by the ingestion of raw or undercooked meat containing *Toxoplasma gondii* cysts or by the ingestion of oocysts from faecally contaminated foods [21,22]. Antibodies against *T. gondii* are found in 10% of the Japanese, 3–35% of the U.S. American, 18–34% of the Spanish and 50–80% of the Central European population [8,21]. Transmission through transplanted organs, including both reactivation as well as newly acquired disease, are possible after Tx [1]. Transmission was mainly observed in seronegative recipients who received grafts from seropositive donors [23]. In this situation, the risk of toxoplasmosis reaches 25–75% in HTx. Myocardial tissue is recognized as a site of toxoplasma cyst deposition [23]. The incidence of toxoplasmosis after BMTx is 0.4–7%, depending of the region. Sixty-nine percent of cases are diagnosed within three months of BMTx [8].

Toxoplasmosis is asymptomatic in 80% of immunocompetent patients. Typical symptoms are lymphadenopathy and fever. The ocular and congenital forms of toxoplasmosis are serious complications in the general population [21]. In immunocompromised patients, a disseminated disease and encephalitis are the most severe presentation of toxoplasmosis. The disseminated disease is mainly observed after BMTx, with mortality above 60% [10,21]. Toxoplasmosis after SOTx is rare. It was reported in 22 out of 15,800 SOTx in Spain [21]. The central nervous involvement in the course of toxoplasmosis is a severe and fatal complication. It can begin with headache. Seizures, hemiparesis, ataxia and cranial nerve palsies may be evident in the later stages. In computed tomography (CT) or nuclear magnetic resonance (NMR) scans, single or multiple hypodense or hypointense contrast-enhancing lesions with mild perifocal oedema in the white matter and basal ganglia may be observed [8,23]. The heart involvement is a severe complication in patients after heart transplantation (HTx). The incidence of post-SOTx toxoplasmosis is closely related to the prevalence of *T. gondii* infection in the general population, which is high in Europe [23]. Risk factors are the consumption of contaminated food and previous cytomegalovirus (CMV) disease [21,24].

Toxoplasmosis is confirmed by the demonstration of *T. gondii* organisms in the blood, body fluids or tissue. Molecular diagnostic methods may also be used for the detection of *T. gondii* DNA in clinical samples. Indirect detection is based on documented seroconversion [21]. Trimethoprim-sulphamethoxazole (TMP-SMZ) used as a prophylaxis for *P. jiroveci* pneumonia is also effective as a prophylaxis for toxoplasmosis and is recommended especially after allo-BMTx in seronegative recipients [21,24].

**Chagas disease** (American trypanosomiasis) is a chronic systemic disease caused by protozoan parasite *Trypanosoma cruzi*. Chagas disease carries high morbidity and mortality in endemic countries [25,26]. It is estimated that there are 10 million people infected with *T. cruzi*, most of them living in Latin America [26]. *T. cruzi* parasites are mainly transmitted by the infected faeces of blood-sucking triatomine bugs, but parasites can also be transmitted by blood transfusion and SOTx. In transplanted patients severe and even fatal form of disease may develop [26]. In endemic countries, Chagas disease is very common (e.g., about 20% of the Bolivian population is infected [27]) but is rarely observed in non-endemic countries. In Europe the transfusion-acquired Chagas disease was reported only in Spain [27]. Asymptomatic *T. cruzi* infection appears to be extremely rare in Europe [26] and the risk of Chagas disease after transplantation is limited.

### 3.5. Protozoan Infections Causing Diarrhoea 

Diarrhoea is one of the most common symptoms after Tx, and is often neglected and regarded by clinicians as an unavoidable side effect of IS implementation. Diarrhoea is observed in 10–30% of patients after Tx [28] and may lead to dehydration, malabsorption, re-hospitalizations, non-compliance with the immunosuppressive regimen and a low quality of life [28]. Possible causes of diarrhoea are drugs (mycophenolate mofetil, tacrolimus, rapamycin, and antibiotics), some metabolic diseases (diabetes) and infections [29]. Bacteria (*Clostridium*, *Campylobacter*, *Shigella* sp., *Salmonella enteritis*), viruses (CMV, norovirus, rotavirus, and adenovirus) and parasites are common agents of infectious diarrhoea [28,30]. In some countries diarrhoea caused by intestinal parasites is common. In Iran 33.3% of KTx recipients are infected with one or more intestinal parasites [31].

**Amoebic dysentery** caused by *Entamoeba histolytica* is a common infection causing bloody diarrhoea in tropical countries. About 400–500 million people are infected, but only 10% of colonized patients are symptomatic [32,33]. Typical presentation is bloody diarrhoea with sharp, crampy postprandial progressive abdominal pain [33]. Approximately 50 million cases of invasive *Entamoeba histolytica* infection are observed annually worldwide, with up to 100,000 deaths [10]. Patients treated with steroids are at increased risk of amoebiasis [33] with severe complications such as liver abscesses [34] or pulmonary amoebiasis [10]. Nevertheless, only few cases of amoebic dysentery after SOTx and BMTx have been reported in the USA and Japan [32,33]. Hand hygiene and avoiding contaminated food and faecal exposure during sexual activity after Tx is recommended in prophylaxis [33].

**Giardiasis**. *Giardia duodenalis* (previously known as *G. lamblia*) is one of the most common cosmopolitan intestinal parasites causing diarrhoea throughout the world [35]. Clinical symptoms of giardiasis may include non-bloody diarrhoea, flatulence, foul-smelling stools, abdominal cramps, nausea, vomiting, fatigue, weight loss or malabsorption [36]. The prevalence of *Giardia duodenalis* infection in the tropics is 30% and 3% of traveller’s diarrhoea is related to giardiasis. Giardiasis is responsible for less than 1% of post-Tx parasite infections. The low incidence is probably caused by the fact that giardiasis is easily treated with metronidazole. Some transplant centres in highly endemic countries recommend screening and treatment of *Giardia duodenalis* infection before Tx [36]. Prevention includes good hygiene and avoiding contact with contaminated water and food [36]. 

**Cryptosporidiosis** is regarded as a frequent cause of traveller’s diarrhoea [37]. The infection is passed via the faecal-oral route from infected hosts, or in contaminated water or food comprising *Cryptosporidium* oocysts [28,37,38]. In immunocompetent individuals diarrhoea is scant and self-limited with duration of 2–26 days [37]. A cryptosporidiosis outbreak was observed in 1993, when more than 400,000 Milwaukee residents became ill and 69 of them died [28]. In patients with HIV infection or after Tx, cholera-like diarrhoea could be present. Other symptoms may include fever, abdominal pain, nausea, vomiting, dehydration, electrolyte imbalance, weight loss or headache [28,37]. In European travellers, newly acquired disease is a typical presentation. In endemic areas like Pakistan, cryptosporidiosis was diagnosed in 53% of patients after Tx with diarrhoea [37].

**Cystoisosporiasis** (isosporiasis) is caused by *Cystoisospora belli* (formerly known as *Isospora belli*), a protozoan parasite distributed worldwide, especially in tropical and subtropical countries [28,39]. In immunocompetent hosts, *C. belli* infection causes a self-limited diarrhoeal illness and severe infection with chronic, life-threatening diarrhoea is uncommon. The disease is caused by the ingestion of oocysts in contaminated material such as fruits, vegetables and water [28]. Diarrhoea due to *C. belli* infection was observed after KTx in India, Turkey and Argentina [39,40,41]. 

**Microsporidiosis**. *Microsporidia* have a worldwide distribution. Almost all cases of human intestinal microsporidiosis are caused by two species, *Enterocytozoon bieneusi* (90%) and *Encephalitozoon intestinalis* (10%) [42]. The means of infection is by ingestion or inhalation of environmentally resistant microsporidia spores, from person to person, from the environment, other mammals or insects [42]. Mild diarrhoea is typical. In patients with IS, disseminated disease could be fatal [42,43,44]. Most cases of human microsporidiosis are associated with different types of immunosuppression, especially with HIV infection [33,41]. Diarrhoea after Tx caused by *E. bieneusi* has been diagnosed in Spain, France, Germany, the Netherlands [44,45] and in a 15-year-old patient after LTx in Poland [46]. Three cases of donor-derived central nervous system microsporidiosis caused by *E. cuniculi* were reported in 2017 in the United States. All patients received organs from one donor. One patient died [47].

### 3.6. Other Parasitic Infections in Transplant Patients

**Babesiosis** is a rare tick-borne zoonosis related to falciparum malaria. *B. microti* and *B. divergens* are the two species most frequently found to infect humans. In the last 40 years, *Babesia microti* has been recognized as an important pathogen in humans in USA [48,49]. In Europe, babesiosis caused by *B. divergens* has been diagnosed mainly in splenectomised patients in former Yugoslavia, France, and the British Isles [48]. Typical symptoms of symptomatic babesiosis are haemolysis with varying degree of severity, fever, jaundice and dark urine [50]. In immunocompromised patients, a severe disease with malaise, high fever, nausea, vomiting, abdominal cramps, headache, myalgias, arthralgia, thrombocytopaenia and renal failure have been reported [49]. There is a risk of transmission with blood transfusion after KTx [49]. One hundred and sixty-two cases of transfusion-transmitted babesiosis were reported in USA between 1980 and 2013 [48].

**Amoebiasis**. Free-living amoebas (FLA) are environmental protozoan parasites that can cause fatal diseases during immunosuppressive treatment. FLA are isolated from soil, sand, ponds, streams, tap water, sea water, physiotherapy pools, and other environmental sources [51,52]. There are three clinical presentations of amoebiasis: granulomatous amoebic encephalitis (GAE), disseminated amoebic disease and amoebic keratitis. In the general population, the problem is keratitis, mostly limited to contact lenses users.

**Granulomatous amoebic encephalitis (GAE)** is a rare, almost always fatal disease. Apart from *Acanthamoeba* spp. other FLA like *Balamuthia mandrillaris* and *Sappinia pedata* may cause GAE. Initial symptoms involve the skin, sinuses or lungs. Multiple recurrent panniculitis-like subcutaneous nodules with eosinophilia, chronic rhinitis and chronic sinusitis are observed [51,52]. The disease becomes rapidly fatal when protozoa cross the blood–brain barrier. Clinical symptoms of GAE are culture-negative fever, cough, nauseas and vomiting, headache, dizziness, vertigo, behavioural changes, lethargy, confusion and other neurological symptoms [51]. GAE was observed mainly after BMTx, but also after SOTx and in HIV-infected patients [51]. Risk factors of GAE are IS, a history of nasal drainage, nasal congestion, exposure to contaminated water or garden soil, and road accidents that result in skin lesions [51,52]. Radiological studies show subarachnoid bleedings, hydrocephalus, ring-enhancing lesions, cortical and periventricular hypodense lesions and oedema. Tissue samples, cerebrospinal fluid examinations, serological tests and PCR could facilitate an early diagnosis. The prognosis is bad. GAE has fatality rate of above 90% [51,52].

**Helminths**. There are plenty of helminths causing infections in people. Helminth infections are still very common in tropical countries; some are common in children in Europe. Fortunately, many gut-dwelling helminths does not lead to morbidity or lab test abnormalities (e.g., enterobiasis). Other infections, e.g., strongyloidiasis or ascariasis, can, in immunocompromised patients, present a life threatening disseminated disease with an uncertain prognosis [53].

**Roundworms (nematodes)**. *Strongyloides stercoralis* is a soil-transmitted intestinal nematode that infects humans by skin penetration [3]. A total of 100–200 million people are infected worldwide and about 50% of cases remain asymptomatic [43]. In endemic tropical and subtropical regions, more than 50% of the population are infected [3]. Strongyloidiasis has rarely been reported in European countries such as Poland or Germany [22,54]. The life cycle of this parasite begin when filariform larvae in contaminated soil penetrate the human host skin and migrate via vessels to the alveoli, then travel via the tracheobronchial route to the mouth, where are coughed up and then swallowed. They mature in duodenum and jejunum [55]. Symptomatic infections typically manifest as GI disorders with diarrhoea and abdominal pain. Sometimes the dermatologic symptoms are present [55].

In patients after Tx, newly acquired disease and the reactivation of latent disease are a serious problem in endemic countries. Transmission has not been reported. There are many cases of strongyloidiasis reported one to six months after SOTx and BMTx in endemic countries like Brazil, Peru and the USA [56], and one case has been reported in Germany [22]. **Hyperinfection syndrome (HIS)** is a severe presentation of disseminated strongyloidiasis observed in immunocompromised patients. The symptoms of HIS are fever, asthenia, anorexia, weight loss, abdominal pain, diarrhoea, GI bleeding, haematuria, dyspnoea, cough or haemoptysis. Progressive pulmonary interstitial pneumonitis with alveolar haemorrhage and respiratory failure are present [55,56]. HIS facilitates the translocation of *Enterobacteriae*, therefore Gram-negative bacilli sepsis is a common complication that worsens the prognosis. Ivermectin is the drug of choice in disseminated disease and HIS. The mortality in patients with HIS is 80–90% [43,56].

**Flukes (trematodes).** Schistosomiasis is the second most prevalent parasitic infection in the world. It affects 200–250 millions of people, mainly in sub-Saharan Africa and causes 20,000 deaths per year [57,58]. People became infected during swimming or wading in freshwater by contact with cercariae, the larvae of the parasite, which penetrate the skin of humans [59]. The main symptoms of early infection, called Katayama syndrome, that occur several weeks after infection are fever, headache, urticaria, myalgias, abdominal pain, and diarrhoea [58,59]. *Schistosoma*, commonly known as blood-flukes, live in the portal and perivescial veins [1]. Therefore, patients with chronic infection presents with urinary or GI symptoms. In advanced stages, the infection leads to severe complications, such as portal hypertension or renal failure [58]. In endemic countries, both newly acquired disease and reinfection are common [58]. Transmission has not been reported [1]. Prognosis after Tx is generally good [59]. Avoiding contact with freshwater in countries in which schistosomiasis occurs is recommended. The drug of choice for treating all species of *Schistosoma* is praziquantel.

**Tapeworms (cestodes)**. Alveolar echinococcosis (AE) caused by *Echinococcus multilocularis* occurs in Northern Europe. The annual incidence of AE in Switzerland is 0.26 cases per 100,000 population. Patients become infected by ingestion of eggs from a definitive host (fox or dog). The larval form (metacestode) produces cysts in the human liver. Normally, the progression is slow and signs of the disease may not be seen for 4–15 years after infection [60]. Advanced AE is an indication for liver transplantation. Reactivation and recurrence with rapid progression due to IS with anti-CD20 was observed after LTx [60]. Cystic echinococcosis (CE) caused by *E. granulosus* has a better prognosis than AE, with a low mortality rate. Three cases of the rapid progression of CE after Tx were reported [60].

**Other tapeworms**. Neurocysticercosis caused by the larval cysts of the pork tapeworm (*Taenia solium*) is one of the most common helminthic infections of the central nervous system [61]. In Europe, a case of neurocysticercosis was reported after Tx [61]. The other tapeworm infections lead to abdominal pain and diarrhoea. Infections with *Hymenolepis nana*, a tapeworm of worldwide distribution with 75 million human carriers [62], and *Diphyllobothrium latum*, a fish-borne parasite, have been reported after Tx [29,63].

**Insects (ectoparasites)**. In Europe, a rare and severe form of *Sarcoptes scabiei* infestation called Norwegian scabies (crusted scabies) was observed after KTx [64,65]. It is a fulminant and highly infectious form due to inability of immune system to control the proliferation of the scabies mite [65]. Crusted scabies is mostly observed in elderly and patients after Tx, with HIV, leukaemia, leprosy or diabetes [64,65]. The bacterial co-infection may lead to brain abscesses in immunosuppressed patients [65]. Without any doubt, the main problem with insects in Europe are vector-borne diseases transmitted by ticks, especially Lyme disease and encephalitis, which are not within the scope of this review [66].

## 4. Discussion

Parasitic infections occur predominantly in the tropics and subtropics. However, a significant increase in tourist travel, transplant tourism, the globalization of the world’s economy and food supply, migration and climate change in the world may change the epidemiology of these diseases [43].

The number of **travellers** is rising each year. Some health problems, such as diarrhoea and fever, are common while travelling in the tropics. The rates of travel-related infections may not increase in patients after Tx, but these patients are at increased risk of developing a severe form of the disease [15]. The majority of patients after Tx do not seek advice before travel [15,67]. Parasites (*Cryptosporidia*, *Giardia* and *Cystoisosporia*) are responsible for 1–20% of traveller’s diarrhoea, which is observed in up to 50% of travellers [15].

The preventive measures include compliance with the hygiene rules, avoiding eating uncooked shellfish and seafood because of the risk of anisakiasis or paragonimiasis infections, avoiding raw or undercooked fish because of the risk of *Diphyllobothrium latum* infection and avoiding uncooked meat because of the risk of *T. gondii, Taenia solium* or *E. hystolitica* infections [15,29]. Protective measures against mosquitoes, sand-flies, ticks and other arthropods are essential [15] to minimalize the risk of malaria and leishmaniasis. In tropical countries, insects are ubiquitous and the use of repellents containing DEET (*N*,*N*-diethyl-3-methylbenzamide) or picaridin, mosquito nets, protective, permethrin-impregnated clothing are also recommended [3]. Travellers should avoid walking barefoot, due to the risk of strongyloidiasis, and swimming in fresh water, due to the risk of *Schistosoma* infection [15].

**Transplant tourism.** Fortunately this is a marginal problem. One of the risk factors of these illegal procedures is donor-derived infection. A Turkish study of 115 transplant recipients transplanted commercially in India, Iraq and Iran reported 10 cases of malaria [68].

The **globalization** of the food supply can cause some public health issues. For example, in Canada in 1996, *Cyclospora cayetanensis* infection related to Guatemalan raspberries caused diarrhoea in approximately 1500 of all those exposed [43]. There is a risk of diseases transmitted by pets. Some parasitic infections are zoonosis, other vectors can live on animals. In Europe, there is a risk of *T. gondii* infection acquired from contact with cats or giardiasis and cryptosporidiosis from dogs [35]. The risk is real, because 60% of Polish families have a cat or dog. *Cryptococcus* oocysts have been found in 1.2–12.5% of dogs and *Giardia* cysts in 6–36% of dogs [35].

**Human migration** is likely to change the epidemiology of parasitic infections [10]. There is a potential risk of transmission of some parasitic diseases with blood and organs [13]. Some infections like amoebiasis or strongyloidiasis are very common in immigrants from endemic countries and an initiation of immunosuppression can trigger the reactivation of some diseases in this group of patients [43]. In the USA, pre-transplant screening of the Hispanic immigrant population for strongyloidiasis, leishmaniasis and *Trypanosoma cruzi* infection is recommended [69].

**New therapies** adversely affecting the immune system can reveal opportunistic infections. There are few interesting examples. Strongyloidiasis is a benign infection, but patients treated with steroids and tacrolimus may develop a fatal hyperinfection syndrome. On the other hand, cyclosporine (CSA) shows some activity against *Strongyloides stercoralis* [57]. Rituximab (RTX), a drug causing “pharmacological splenectomy”, is theoretically a risk factor for babesiosis. In Europe, fatal cases of babesiosis were observed only in patients after surgical splenectomy [70].

## 5. Conclusions

Solid organ and bone marrow transplantations, blood transfusions and immunosuppressive treatment are associated with a small but real risk of parasitic infections in European citizens. In the case of severe infections (disseminated disease, brain or lung involvement) the progression is very rapid and the prognosis is bad. Establishing the diagnosis before the patient’s death is challenging.

## Figures and Tables

**Table 1:** Endemic areas, species of pathogens, diagnosis and treatment of presented diseases.

Disease	Parasite	Endemic Areas	Diagnosis	Treatment
Malaria	*Plasmodium falciparum*, *P. malariae*, *P. ovale*, *P. vivax*, *P. knowlesi*	Tropical	Microscopic diagnosis (thick and thin blood smears); rapid diagnostic tests for malarial antigen; PCR assay testing	**Uncomplicated malaria**: *P. falciparum*—atovaquone with proguanil or artemether with lumefantrine *P. vivax*, *P. ovale*—chloroquine phosphate with primaquine phosphate **Severe malaria**: artesunate or quinine iv with doxycycline or clindamycin.
Visceral leishmaniasis	*Leishmania donovani*	Tropical and temperate regions in Africa, China, India, Nepal, South America, southern Europe	Microscopic examination of bone marrow, peripheral blood or liver aspirate; PCR assay testing; serology	**Systemic therapies**: pentavalent antimonial compounds (sodium stibogluconate), pentamidine, liposomal amphotericin B, paromomycin, miltefosine **Alternative treatment**: fluconazole, itraconazole, ketoconazole, allopurinol, dapsone. **Local therapies**: cryotherapy, infiltration of sodium stibogluconate, topical paromomycin or urea
	*L. infantum*, *L. chagasi*	Mediterranean countries, Latin America		
Dermal leishmaniasis	*L. tropica*, *L. major*	The Middle East, China, India, Pakistan, Africa, Mediterranean countries	Microscopic examination of skin lesion specimens; PCR assay testing	
	*L. aetiopica*	Africa (Ethiopia, Kenya, Yemen)		
	*L. mexicana*, *amazonensis*, *brazilensis* et al.	America		
Toxoplasmosis	*Toxoplasma gondii*	Cosmopolitan	Microscopic examination of infected tissue, blood or CSF; serology; PCR assay testing; imaging studies; fundoscopic examination	Pyrimethamine + folinic acid + sulfadiazine or clindamycin. **Alternative treatment**: pyrimethamine + folinic acid +azithromycin or clarithromycin or atovaquone or dapsone or trimethoprim/sulfamethoxazole **Pregnant women**: spiramycin, pyrimethamine + folinic acid + sulfadiazine
Babesiosis	*Babesia microti*	Europe	Microscopic diagnosis (thick and thin blood smears); PCR assay testing; serology	Quinine + clindamycin Atovaquone + azithromycin
	*B. divergens*	USA		
GAE	*Acanthameba* sp. *Balamuthia mandrillaris*	Cosmopolitan	Histological examination of biopsied tissue; serology; PCR assay testing	Surgical resection with medical treatment. Amphotericin, miltefosine, rifampicin, fluconazole, azithromycin, pentamidine + sulfadiazine
Amoebiasis	*Entamoeba histolytica*	Tropical Subtropical	Stool examination; histological examination of biopsied tissue; serology	Metronidazole, tinidazole Luminal amebicide: Paromomycin, diloxanide furoate
Giardiasis (lambliosis)	*Giardia duodenalis*	Cosmopolitan	Stool examination; duodenal biopsy and aspirates	Metronidazole, albendazole, nitazoxanide
Cryptosporidiosis	*Cryptosporidium* sp.	Cosmopolitan	Stool examination	Nitazoxanide
Cystoisosporiasis	*Cystoisospora belli*			Trimethoprim/sulfamethoxazole, pyrimethamine, ciprofloxacin
Cyclosporiasis	*Cyclospora cayetanensis*			Trimethoprim/sulfamethoxazole
Microsporidiosis	*Enterocytozoon bieneusi* *Encephalitozoon intestinalis*	Cosmopolitan	Stool and urine examination; PCR assay testing; histologic examinations	Topical and oral fumagillin Albendazole
Strongyloidiasis	*Strongyloides stercoralis*	Cosmopolitan, endemic in the tropics and limited foci in USA, Europe, Australia and Japan	Stool examination; serology	Ivermectine, albendazole
Schistosomiasis	*S. japonicum*	The Far East	Stool or urine examination; serology; histological examination of biopsied tissue	Praziquantel
	*S. mansoni*	The Middle East, Western Africa, South America		
	*S. haematobium*	Africa, The Middle East, India		
Taeniasis	*Taenia solium*, *T. saginata*, *T. asiatica*	Cosmopolitan	Stool examination; serology	Praziquantel, albendazole
Cysticercosis	*T. solium*	Cosmopolitan	Imaging studies	Praziquantel, albendazole; surgery
Hymenolepiasis	*Hymenolepis nana*	Cosmopolitan	Stool examination	Praziquantel, nitazoxanide, niclosamide
Alveolar echinococcosis	*Echinococcus multilocularis*	Northern hemisphere	Serology; imaging studies	Albendazole, mebendazole, surgery (partial resection of a liver, hemihepatectomy, liver transplantation), biliary stenting
Cystic echinococcosis	*Echinococcus granulosus*	Cosmopolitan		Albendazole, mebendazole, surgery (total pericystectomy or partial hepatectomy), percutaneous treatment (PAIR)
Norwegian scabies	*Sarcoptes scabiei hominis*	Cosmopolitan	Microscopic visualization of mites, larvae, ova or feces in skin scrapings	Ivermectin, permethrin, lindane
Demodicosis	*Demodex folliculorum*	Cosmopolitan	Microscopic visualization of *Demodex* mites in skin scrapings	Crotamiton cream, permethrin cream, topical or systemic metronidazole, ivermectin

**Table 2:** The most important parasitic infections after transplantation in Europe.

Disease	Type of Transmission			Importance	
	Donor Derived	Reactivation	New Onset	Population in Danger	Mortality
**Malaria**	Possible, few cases were reported in Europe	Possible in *P. vivax* and *P. ovale* infections	The most common	One of the most important causes of fever in travellers to tropics	6% mortality after KTx, 40% mortality after LTX.
**Visceral leishmaniasis**	Suggested in literature	Possible	The most common	Travelers to tropics, inhabitants of South Europe	20% mortality after Tx.
**Toxoplasmosis**	Possible, serological examination of donors is recommended	The most common	Possible	High prevalence in Europe	60% mortality in disseminated disease.
**Amoebiasis**	Unlikely	Possible	Possible	Travelers to tropics	High mortality in disseminated disease. Liver amoebic abscess is a typical complication in patient with IS.
**Other protozoan infections causing diarrhoea**	Unlikely, although cases of donor derived microsporidosis were reported	Possible	Possible	Giardiasis is relatively common in Europe. The real danger is a possiblity of epidemic caused by contaminated food or water.	Relatively mild when only diarrhoea occurs. High mortality in disseminated disease.
**Strongyloidiasis**	Not reported	Possible	Possible	Travelers to tropics.	HIS is a typical manifestation of disseminated disease with high mortality.
**Schistosomiasis**	Not reported	Possible, especially when schistosomiasis was an indication for LTx	Possible	Travelers to tropics	Relatively good prognosis with low mortality.
**Infections caused by tape worms**	Unlikely	Possible, especially when AE was an indication for LTx	Possible	Rare but still present in in Europe	Relatively good prognosis with low mortality.
